# Comparing AI-Assisted Coding and Traditional Qualitative Analysis: a Study Examining Differences in Methods and Results of AI-Assisted Coding and Traditional Content Coding Using Community Engagement Data Collected During the Development of a Municipal Food Plan

**DOI:** 10.1007/s11121-026-01904-4

**Published:** 2026-03-26

**Authors:** Natalie Poulos, Hannah Price, Lauren Bell, Courtney Byrd-Williams, Sergio Torres-Peralta, Edwin Marty

**Affiliations:** 1 Department of Nutritional Sciences, University of Texas at Austin, Austin, TX USA; 2 School of Public Health, UT Health Science Center Houston, Austin Campus, Austin, TX USA; 3 City of Austin, Communications and Public Information Office, Austin, TX USA; 4 City of Austin, Austin Climate Action and Resilience, Austin, TX USA

**Keywords:** Artificial intelligence, AI-assisted coding, Qualitative analysis, Content analysis, GPT

## Abstract

Qualitative data poses a challenge for prevention science and public health, as it is critical to explain the context of communities, health, and behavior, yet collecting and analyzing qualitative data using traditional methods is time-intensive and requires extensive training. As artificial intelligence (AI) models have improved, there is a growing interest in using AI to code qualitative data quickly and reliably. This study compares the similarities and differences in methods and results of artificial intelligence (AI)-assisted qualitative analysis to traditional qualitative content analysis using data collected during the development of a city and county-based food plan. In total, 2820 community comments were collected across 43 community events in 27 zip codes across the region between March 2023 and January 2024. AI-assisted analysis was completed using a combination of a transcription app (Post-It^Ⓡ^), GPT4 Plus, and GPT for Sheets with oversight from a public health practitioner. Traditional qualitative content analysis was completed with two trained coders who completed codebook development, reliability analysis, and full content coding. Both methods used deductive codes to represent key aspects of the food system and generated inductive codes to represent areas not included by the deductive food system codes. Results found that AI-assisted methods and traditional content analysis produced similar deductive coding results, while inductive coding results were less comparable across methods. Given that qualitative data has become a central part of prevention science, we believe with careful considerations, AI-assisted methods with intentional oversight have the potential to strengthen our ability to process large amounts of qualitative data.

**Supplementary Information:**

The online version contains supplementary material available at https://doi.org/10.1007/s11121-026-01904-4.

## Introduction

Traditional qualitative analysis is the dominant method of analyzing non-numeric data that describes and contextualizes human behavior and experiences (Sofaer, 1999). Qualitative data collection has become a key component of prevention science efforts because of the emphasis on understanding the context of health, disease, and behaviors within a community (Jeffries et al., 2019; National Cancer Institute, 2020). Although qualitative data is often collected in the form of interviews and/or focus groups, it is often underused because of the time, training, and cost it takes to complete reliable analysis of qualitative data (Prescott et al., 2024). Given the importance of understanding community context within prevention science, there is a need for more efficient qualitative data collection and analysis methods that maintain analytic rigor.

Artificial Intelligence (AI) is a rapidly changing field of technology that has the potential to be used to efficiently identify, interpret, and structure data in multiple facets (Centers for Disease Control and Prevention, 2023; Wang et al., 2022). To date, AI has already proved effective in medical diagnosing, medical documentation, contact tracing, drug formulation, and public health surveillance (Olawade et al., 2023; Xiao et al., 2023). Recent research suggests AI may expedite the process of qualitative data analysis through the use of large language models (LLM), such as generative pre-trained transformer (GPT), which use deep learning architectures to understand and generate human-like text. Research suggests that these machine learning models can perform natural language processing to organize textual data into content and thematic codes (de Paoli, 2023; Prescott et al., 2024), yet many questions remain on the effectiveness and appropriateness of LLMs for qualitative analysis. For example, three recent studies compared AI-assisted coding to traditional qualitative coding. All studies used LLM platforms, such as GPT-3.5, GPT-4, or ChatGPT, and compared them to qualitative thematic analysis. Results found that they can successfully be used to code simple responses (Gamieldien et al., 2023), focus groups (Morgan, 2023), and interviews (Li et al., 2024) similarly to traditional thematic analysis, yet authors all suggest additional research is needed to fully understand the usefulness and techniques needed to effectively code qualitative data in a meaningful way. It should be noted that all studies focus on inductive forms of analysis where AI was asked to interpret text and generate themes, but did not ask AI to match text to codes identified a priori. Lastly, a known shortfall of using AI to code qualitative data is the validity of AI software or LLMs to accurately reflect the context and meaning of qualitative data (Prescott et al., 2024). Nevertheless, AI-assisted coding may be useful when managing large amounts of data due to the rapid processing speed.

Given the advances of AI-assisted qualitative analysis and the need for more efficient qualitative analysis methods, this study explores the use of AI in qualitative content analysis by comparing AI-assisted coding to traditional qualitative analysis. The research question guiding this investigation is: *What are the similarities and differences between AI-assisted qualitative coding with public health practitioner oversight and traditional content analysis completed by trained researchers?* We explore the prominent codes identified by each method and the accuracy of the AI in interpreting nuanced qualitative data using a dual analytical strategies of deductive and inductive coding. By comparing these two approaches and coding strategies, we aim to understand how AI tools can complement or improve upon traditional qualitative research methods.

## Methods

### Study Context

In September 2021, the City of Austin, TX and Travis County (ATC) issued a directive to create the first comprehensive Food Plan for ATC (City of Austin, 2024). The Food Plan was to include goals and strategies to create a more equitable, resilient, and sustainable food system for all residents of ATC. To ensure the success of a co-created Food Plan, multiple strategies were implemented, including a comprehensive assessment of the current food system, the development of a Community Advisory Committee, community engagement events, and community workgroups focused on food production, food access, markets and retail, processing and distribution, and food recovery. This study leverages qualitative community engagement data collected for the development of the ATC plan. This study was reviewed by The University of Texas at Austin Institutional Review Board and determined to not be human research, as all data was collected voluntarily and anonymously during public engagement for local government (STUDY00005681).

Community engagement data was collected by tabling at community events, city-organized events, and online surveys to identify key concerns that the community had about the current food system. At the tabling and organized events, a city staff member described the upcoming food plan development, the importance of community engagement, and then asked if the individual was interested in participating by answering two key questions: (1) What are your hopes and dreams for the Austin/Travis County food system? and (2) What are the challenges and concerns you have for the Austin/Travis County food system? Community members were asked to write their responses on sticky notes that were then pasted to a poster board on the table. Online surveys were available through the public input website managed by the city and asked community members to respond to the same two questions focused on “Hopes & Dreams” for the food system and “Challenges & Concerns” within the current food system. These questions were chosen, as this phase of the food plan development was focused on visioning. All data was collected anonymously and voluntarily between March 2023 to January 2024.

In total, 2820 qualitative comments were collected from community members across 43 community events located in 27 different zip codes. Events were targeted by zip code to provide equitable representation across multiple groups and communities living in the City of Austin and Travis County. For example, events were planned in zip codes with known target populations. These populations included the American Indian or Alaskan, Asian and Asian American, Black and African American, General Population, Hispanic or Latinx, Sexual and Gender Minority groups, Migrant or Immigrant, Seniors (60 years or older), College Student, Unsheltered or Unstable Housing, Women, and Youth (aged 18–21). Given that participation was voluntary and anonymous, total engagement at each event was not documented under the assumption it would deincentivize participation and result in less participation.

### Digitizing Community Comments

**Fig. 1:**
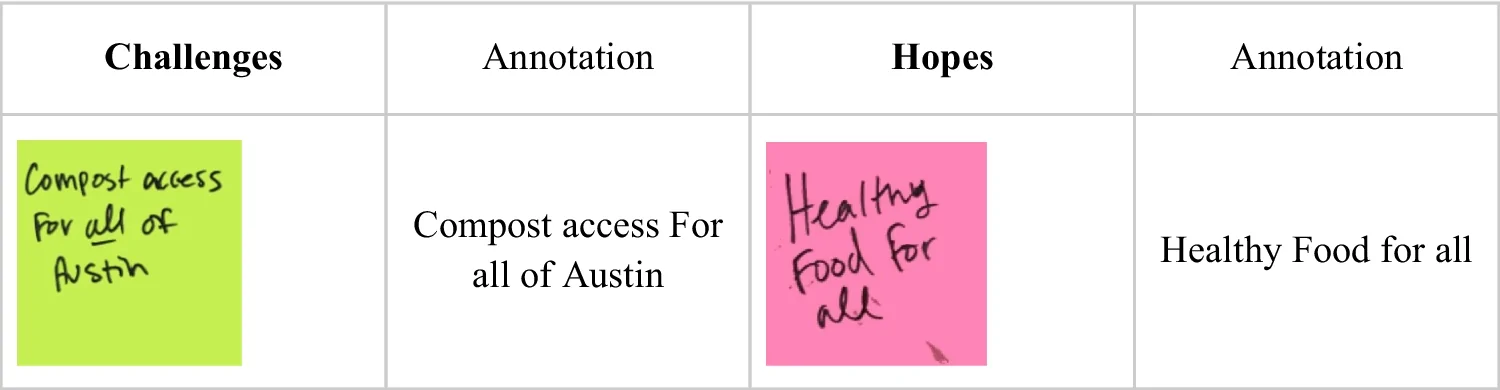
Field post-it note to digitized comment using the Post-It^Ⓡ^ application

After each community event, each hand-written post-it note was digitized using an app called Post-It^Ⓡ^ (https://www.post-it.com/), a mobile application that used the camera of a smartphone to document content on unique post-it notes and convert it into digital text. Each digitized response represented a unique qualitative response from a community member. Digitized community comments were then exported via Post-It^Ⓡ^ app to Google Sheets, where comments were organized on individual rows in two arrays, one array for “Hopes & Dreams,” and another array for “Challenges & Concerns.” Results from the online survey, which included the same prompts, were exported and added to the same two arrays in Google Sheets. See Fig. 1 for an example of how a community-written comment was digitized.

### Coding Strategy

Qualitative analysis of all 2820 community responses was completed using two different methods: (1) AI-assisted analysis using GPT for Sheets powered by ChatGPT for Work, with public health practitioner oversight, and (2) traditional qualitative content analysis was completed by researchers. The Standards for Reporting Qualitative Research was used to document necessary content (O'Brien et al., 2014).

Both strategies of analysis used deductive and inductive coding to describe the content of community comments. Deductive codes included codes that were identified and priority issue areas in a previous report from the City of Austin in the State of the Food System Report (City of Austin & Office of Sustainability, 2022). These included codes focused on the food system and included the following: Markets & Retail, Processing & Distribution, Access & Consumption, Food Production, and Post Consumption & Food Waste. An additional code of “Not Applicable or N/A” was also included to identify comments that did not fit into a deductive code. Definitions of deductive codes used by both types of analysis can be found in Table 1.

**Table 1:** Food system code definitions and key terms used in deductive analysis

Codes	Definition of code^1^	Example key terms^2^
Markets & Retail	How food is sold and purchased	Grocery stores, bodegas, farmers markets, food jobs, restaurants, food apps, workers rights, selling food
Processing & Distribution	What happens to food from where it is grown to when it reaches your plate, including how food is moved and processed	Food processing facilities, food permits, food regulations, food aggregation, food distribution, supply chain, food delivery, food hubs, truckers warehouses, anything related with how food travels
Access & Consumption	How we eat our food who struggles to get enough food, and what impact our consumption has on our health	SNAP, hunger relief programs, prices of food, hunger related policy, location of grocery stores, accessibility, disability access, access to transportation, nutrition, health, wellness, racial and ethnic disparities, alternative diets, homelessness, discrimination
Food Production	Where our food comes from, including everything from farming to ranching to backyard gardening	Farming, agriculture, farms and environmental impact, green jobs, environmental sustainability, urban farms, land ownership, ranching and cattle, incentives for farming
Post Consumption & Food Waste	What happens to the parts of food we do not eat and the impact of food waste on the environment	Food Recovery Apps, Composting, Trash pickup, Resource Management, Waste Prevention, Smart purchasing, Better purchasing practices, food rescue
N/A	Not applicable or not relevant	Anything that does not quite fit

^1^Definition identified in State of Food System Report

^2^Key terms used for both AI-assisted and traditional qualitative coding

Inductive codes were also generated by each analysis method. Inductive codes were created to represent overarching content areas. These codes were unique to each method of analysis, both in method and content.

### AI-Assisted Analysis

GPT for Work (https://gptforwork.com/), an AI-powered tool that is available as an extension for Google Sheets (https://docs.google.com/spreadsheets), was used to map qualitative community comments to identified codes. GPT-4 Plus (https://openai.com/index/gpt-4/) was used to support theme generation. In this study context, “theme” was used to highlight groupings of codes that represented a broader content area. This approach leverages AI capabilities to identify patterns and assign thematic categories to qualitative data. Each step of mapping comments to deductive codes and the creation of inductive codes is described in detail below.

#### Step 1: Mapping Community Comments to Deductive Food System Codes

Using GPT extension for sheets, all community comments within a single array were then mapped to deductive food system codes. This required a formula that included the search key (deductive codes), array of data to be included, and the confidence threshold of the analysis to be included. The formula used to complete mapping was GPT_MAP (Search_keys, Data, Confidence level, Display stats, Top Results). Search keys represented the deductive code (e.g., Markets & Retail) with the key terms identified before analysis. These terms served as the lexicon for GPT for Work to organize the data. “Data” selects the array of data to be coded, “confidence level” is determined by user from 0 to 1 (0 = no confidence, 1 = high confidence), “display stats” allows for inclusion of confidence values, and “top results” allows for results to be capped at a specific number of matches. After executing the GPT_MAP function, GPT for Sheets then outputs columns that filter each comment based on how closely it relates to the search term (deductive code). Based on a review of mapped results by the city and county staff who participated in data collection, the confidence for mapping individual comments to deductive codes was set to 0.7966, or a confidence of 79.66% that GPT for Sheets was accurately matching the qualitative comment to the deductive code. Mapping comments is the process that GPT for Sheets uses to match each comment to the identified deductive code. For example, GPT for Sheets considered each community comment and determined with 79.66% confidence which deductive food system code that comment best matched. If GPT for Sheets was not at least 79.66% confident that the community comment matched the deductive food system code, the comment was not coded. This confidence level was determined through trial and review by the public health practitioner of all matched results to ensure an appropriate level of matching of comments to deductive codes, while also minimizing irrelevant matches. The practitioner set varying levels of confidence, ran the GPT_MAP command, and reviewed how comments were mapped to codes. After multiple iterations, 0.7966 was determined to be the most appropriate and was used for all MAP commands.

Mapping was completed on each array, “Hopes & Dreams” and “Challenges & Concerns,” separately. The reduction in confidence from 1.0 to 0.7966 resulted in 355 comments not being matched to a deductive code among the array of “Hopes & Dreams” and 557 comments not being matched to a deductive code among the array of “Challenges & Concerns.” An example of a comment from the “Hopes & Dreams” array that was not mapped includes “I hope butterflies will come.” An example of a comment from the “Challenges & Concerns” array that was not mapped includes “Too many preservatives.” This reduction in total comments was due to AI not having the confidence to match a comment to a code. After review of some of the comments dropped, the most common reasons were irrelevant codes that were not related to the food system or transcription errors that made a comment illegible.

#### Step 2: Identifying Themes

To identify additional content areas and themes that were not represented by the deductive food system codes, a series of prompts within GPT-4 Plus were used to begin generation of themes. Community comments were pasted into GPT-4 Plus with the following prompt, “Analyze the following comments and provide me with the top themes related to food” and “Find overarching themes across all of the following comments related to the draft of the Food Policy Plan for Austin and Travis County. In your answer, provide the count of how many comments relate to each overarching theme.” These preliminary outputs served to test the analysis capacity of GPT and to identify the best way to ask the prompt. Later, GPT 4-Plus was asked, “Find the underlying themes for each of the following prompts, provide your output in a table format.” This resulted in hundreds of themes, with 1–5 themes attached to a single comment. This preliminary output was then copied to Google Sheets for further analysis using GPT for sheets. To refine the identified themes, GPT for Sheets was then prompted to “Summarize the following array to 25 different themes, output at a table.” After reviewing the 25 generated themes, it was determined that another refinement was necessary to allow for interpretability of the data overall. GPT for Sheets was then prompted to “Summarize the following array to 12 different themes, output as a table.” This resulted in an output that was identified as realistic, authentic, and representative of the data. To ensure that GPT for Sheets was grouping data in a way that was relevant to the project, the AI-software was prompted to create definitions of each theme using the prompt “Thank you; Please include the definition on a separate column.” This resulted in 12 created themes.

To ensure that themes authentically represented the data and experience of community members, the public health practitioner in discussion with colleagues who had participated in data collection reviewed all themes. All generated themes were discussed to ensure alignment with data collection experiences and conversations. After discussion between the public health practitioner completing the data analysis and two additional practitioners who participated in data collection, some themes were rearranged and/or expanded to better mirror community voice. In addition, three themes were added to represent comments that practitioners deemed unrepresented by the current themes. In total, 15 total themes were used in thematic analysis.

#### Step 3: Mapping Themes to Community Comments

Finalized themes were then mapped to community comments to identify the extent to which each theme was represented in the data. This was completed similarly to mapping deductive food system codes using the formula, GPT_MAP (Search_keys, Data, Confidence level, Display stats, Top Results). Similar to mapping deductive food system codes, thematic mapping was completed on each array, “Hopes & Dreams” and “Challenges & Concerns,” separately. The confidence was set to 0.792 or 79.2% as determined by the public health practitioner. This resulted in 328 comments not being mapped to the “Hopes & Dreams” array and 459 comments not being mapped to the “Challenges & Concerns” array.

#### Traditional Qualitative Analysis

Reflexive content analysis was chosen to as the primary data analysis method, as it is a widely used method for examining simple qualitative data such as brief, context-limited comments (Nicmanis, 2024). Reflexive content analysis also supports the identification of underlying concepts within qualitative datasets (Elo & Kyngas, 2008). Two types of codes were used during reflective content analysis, deductive codes and inductive codes. Deductive codes represented food system areas that were predetermined based on the State of the Food System Report (City of Austin & Office of Sustainability, 2022) published by the Office of Sustainability, City of Austin, including Food Processing and Distribution, Food Access and Consumption, Food Markets and Retail, Post-consumption and Food Waste, and Food Production. Inductive codes were created after a complete review of community comments to represent categories of overlapping codes that were not represented by the deductive food system codes. Detailed list of coding steps are provided below.

All coding was completed by two individuals with backgrounds in nutrition and public health. Both researchers also had experience with data analysis and coding qualitative data. Neither coder was involved directly with data collection. In addition to two coders, two additional public health researchers with qualitative data analysis experience supported codebook development and analysis planning. Both primary coders were blinded to existing thematic codes created in the AI-assisted analysis.

##### Step 1: Creation of Codebook

A codebook was created and included deductive codes that were based on the State of the Food System Report 2022, the most up-to-date assessment of the Austin & Travis County Food system at the time. These deductive codes were the same deductive codes used in the AI-assisted analysis. The codebook outlined definitions, key works, and what a code was not meant to include for each deductive code.

##### Step 2: Import Data for Analysis

All digitized comments were imported into Google Sheets as two separate arrays, “Hopes & Dreams” that included 1518 comments and “Challenges & Concerns” that included 1302 comments. Arrays were housed in separate sheets within the same Google Sheet document, with each comment represented on a unique row in the sheet. Deductive codes were documented as headers of columns.

##### Step 3: Test Inter-Rater Reliability

Inter-rater reliability (IRR) was tested in two waves. The first test of IRR focused on deductive codes. A 13–15% sample of the community engagement comments was selected from each array, Hopes & Dreams (*n* = 200) and Challenges & Concerns (*n* = 199). Cells that were identified as corresponding to the criteria set by the row (community comments) and column (deductive codes) were marked in the appropriate matched cell via the number “1” if the comment fit the respective code. After the completion of this first round of coding, the coders reviewed the coding sample and discussed discrepancies. After discussion, the codebook was refined, and a series of inductive codes were added to the codebook. These inductive codes represent sentiments that could not be categorized effectively into prior deductive codes.

The second test of reliability was completed after the codebook was finalized to include adjustments to deductive code definitions as well as the addition of new inductive codes. A second random sample of 200 comments was selected from both the “Hopes & Dreams” array and “Challenges & Concerns” array. The same two coders separately completed coding and then compared results. The percent agreement of the finalized codebook ranged from 86 to 100% agreement across all codes. When measured using Cohen’s Kappa, moderate agreement was found for both Hopes & Dreams (*κ* = 0.50) and Challenges & Concerns (*κ* = 0.53). A detailed report of the percent agreement according to each code and array can be found in supplemental materials, Supplemental Table 1.

##### Step 4: Code All Comments

Using the finalized codebook, all 2820 comments were coded by the same two coders. One coder completed coding on the “Hopes & Dreams” array, while the other completed coding on the “Challenges & Concerns” array.

To provide consistency and transparency in the qualitative data analysis and reporting procedures, the Standards for Reporting Qualitative Research checklist was completed and included in the Appendix, Table 7. The complete work flow of each coding step for both AI-assisted and traditional qualitative analysis can be found in Supplemental Fig. 1.

### Data Analysis

To compare the similarities and differences of AI-assisted coding and traditional qualitative analysis, frequencies and ranks of deductive codes were compared by analysis method and array. Due to differences in methods, inductive codes were not compared, but the description and rank of codes were reported. All coding results were exported to Microsoft Excel for data analysis.

## Results

### Deductive Analysis of Food System Codes

#### AI-Assisted Food System Coding

Food consumption and access were the top food system codes for both “Challenge & Concerns” (37%,* n* = 271) and “Hopes & Dreams” (28%, *n* = 332), while food processing and distribution were the least used codes across both arrays, 8% of comments within “Challenges & Concerns” and 4% of comments within “Hopes & Dreams.” Within “Challenges & Concerns,” after consumption and access, the next top concern was food waste (21%, *n* = 152), followed by food markets and retail (20%, *n* = 151), and food production (15%, *n* = 108). After consumption and access within “Hopes & Dreams,” food markets and retail was the next top concern (25%, *n* = 288), followed by food production (24%, *n* = 276), and then food waste (19%, *n* = 226). Due to the confidence interval, 557 (43%) comments were not coded in the “Challenges & Concerns” array, while 355 (23%) were not coded from the “Hopes & Dreams” array. See Table 2.

**Table 2:** AI-Assisted coding prevalence of food system codes (deductive) by data array, Challenges & Concerns (*n* = 1302) and Hopes & Dreams (*n* = 1518)

AI-assisted analysis counts	Challenges & Concerns		Hopes & Dreams	
Food system codes	*n*	%	*n*	%
Food Production	108	15%	276	24%
Food Consumption/Access	271	37%	322	28%
Food Markets/Retail	151	20%	288	25%
Food Waste	152	21%	226	19%
Food Processing/Distribution	58	8%	49	4%
Not Applicable	5	0%	2	0%
Did not fit a food system code	557	43%	355	23%
Total	1302	100%	1518	100%

#### Traditional Qualitative Food System Coding

Within the “Challenges & Concerns” array, the top food system code was food consumption and access (32%, *n* = 184), followed by food waste (27%,* n* = 152), food production (22%, *n* = 128), markets and retail (15%, *n* = 85), and food processing and distribution (4%, *n* = 21). Within the “Hopes & Dreams” array, the top food system code was food production (37%, *n* = 282), followed by food consumption and access (33%, *n* = 251), food waste (17%, *n* = 133), food markets and retail (9%, *n* = 71), and food processing and distribution (4%, *n* = 29). Within the “Challenges & Concerns” array, 556 (43%) comments were not coded as a comment related to a food system code. Within the “Hopes & Dreams” array, 685 (45%) comments were not coded as a comment related to a food system code. See Table 3.

**Table 3:** Reflexive content analysis coding prevalence of food system codes (deductive) by data array, Challenges & Concerns (*n* = 1302) and Hopes & Dreams (*n* = 1518)

Traditional qualitative analysis counts	Challenges & Concerns		Hopes & Dreams	
Food system codes	*n*	%	*n*	%
Food Production	128	22%	282	37%
Food Consumption/Access	184	32%	251	33%
Food Markets/Retail	85	15%	71	9%
Food Waste	152	27%	133	17%
Food Processing/Distribution	21	4%	29	4%
Not Applicable	176	14%	67	4%
Did not fit a food system code	556	43%	685	45%
Total	1302	100%	1518	100%

#### Ranking of AI-Assisted Results v. Traditional Qualitative Results for Food System Codes

Rank of codes was determined by the total count of codes within each deductive food system code. Based on deductive coding, AI-assisted analysis and qualitative analysis identified the same top concern of community members as “Food Consumption & Access” and the same bottom concern as “Food processing & Distribution.” Other food system codes ranked differently across AI-assisted and qualitative analysis. See Table 4 for ranks of AI-assisted food system codes compared to qualitative analysis food system codes.

**Table 4:** Rank of AI-assisted coding compared to traditional qualitative analysis coding for food system codes

Coding rank	AI-assisted		Qualitative analysis	
	Challenges & Concerns	Hopes & Dreams	Challenges & Concerns	Hopes & Dreams
1 (top)	Food Consumption & Access*	Food Consumption & Access*	Food Consumption & Access*	Food Production
2	Food Waste*	Food Markets & Retail	Food Waste*	Food Consumption & Access
3	Food Markets & Retail	Food Production	Food Production	Food Waste
4	Food Production	Food Waste	Food Markets & Retail	Food Markets & Retail
5 (bottom)	Food Processing & Distribution*	Food Processing & Distribution*	Food Processing & Distribution*	Food Processing & Distribution*

*Bolded represents food system codes that were ranked the same between analysis methods

### Inductive Coding Results

#### AI-Assisted Inductive Analysis

AI-assisted inductive analysis suggests that the top theme among both the “Challenges & Concerns” array and “Hopes & Dreams” array was access to food and affordability (31%, *n* = 715 for Challenges & Concerns; 25%, *n* = 932 for Hopes & Dreams) closely followed by agriculture and local food production (24%, *n* = 546 for Challenges & Concerns; 25%, *n* = 908 for Hopes & Dreams). Nutrition, Health, and Wellness were the next most common inductive code across both arrays (9%, *n* = 217 for Challenges & Concerns; 10%, *n* = 371 for Hopes & Dreams). Community engagement was a common code among the “Hope & Dreams” array (10%, *n* = 375), but was less frequent in “Challenges & Concerns” (5%, *n* = 106). All other codes represented less than 7% of all coded comments. See Table 5 for additional detail of inductive codes.

**Table 5:** AI-assisted analysis of inductive coding prevalence by data array, Challenges & Concerns (*n *= 1302) and Hopes & Dreams (*n *= 1518)

Inductive Codes	Challenges & Concerns		Hopes & Dreams	
	*n*	%	*n*	%
Access to Food & Affordability	715	31%	932	25%
Agriculture & Local Food Production	546	24%	908	25%
Nutrition, Health, & Wellness	217	9%	371	10%
Community Engagement	106	5%	375	10%
Sustainability & Environment	134	6%	229	6%
Resource Management & Waste Prevention	136	6%	153	4%
Access to Land & Home Ownership	121	5%	227	6%
Transportation & Infrastructure	92	4%	78	2%
Education, Innovation, & Resource Building	47	2%	139	4%
Justice & Social Disparities	73	3%	64	2%
Advocacy & Civic Engagement	33	1%	118	3%
Not Applicable	24	1%	14	0%
Resilience & Emergency Response	14	1%	22	1%
Diversity, Faith, & Cultural Inclusion	16	1%	34	1%
Economic Issues & Labor	20	1%	13	0%
Policy & Government Action	10	0%	13	0%

#### Traditional Qualitative Inductive Coding

The most common inductive codes within the “Challenges & Concerns” array were cost and income (21%,* n* = 232) and food quality (20%, *n* = 218). The next most common inductive code was nutrition education (8%, *n* = 90). All other codes within the “Challenges & Concerns” array represented less than 8% of community comments.

The most common inductive code within the “Hope & Dreams” array was food quality (22%, *n* = 202). The next most common inductive code was nutrition education (12%,* n* = 111), community (10%, *n* = 86), and cost and income (9%, *n* = 79). All other codes within the “Hopes & Dreams” array represented less than 8% of community comments. See Table 6 for complete prevalence of all codes identified during qualitative content and thematic analysis.

**Table 6:** Traditional qualitative analysis of inductive coding prevalence by data array, Challenges & Concerns (*n* = 1302) and Hopes & Dreams (*n* = 1518)

Inductive Codes	Challenges & Concerns		Hopes & Dreams	
	*n*	%	*n*	%
Food Justice	18	1.61%	41	4.55%
Schools	35	3.13%	57	6.32%
Nutrition Education	90	8.04%	111	12.31%
Inclusive + Culturally Relevant Foods	40	3.57%	70	7.76%
Food Policy	60	5.36%	50	5.54%
Environmental Impact	57	5.09%	45	4.99%
Transportation	62	5.54%	40	4.43%
Food Quality	218	19.48%	202	22.39%
Health	41	3.66%	17	1.88%
Cost/Income	232	20.73%	79	8.76%
Housing	40	3.57%	37	4.10%
Community	50	4.47%	86	9.53%

## Discussion

Overall, AI-assisted qualitative coding with oversight from a public health practitioner produced similar results to traditional qualitative coding completed by two trained researchers. Both analyses identified the same top and bottom priority among deductive food system codes. This overall similarity is echoed by previous research that compared qualitative coding across AI platforms and manual techniques (de Paoli, 2023; Gamieldien et al., 2023; Prescott et al., 2024). Yet, this analysis did suggest that there are likely still differences in how AI-assisted methods and traditional qualitative methods would rank all codes included in the analysis.

Similarities in deductive coding is evidenced by the ranking of similar deductive food system codes as the top-ranked food system codes and lowest ranked food system codes. This is reasonable as deductive analysis frameworks are more feasible as they are pattern-based, and AI can pull data around certain codes and appropriately apply information to coding. While coding methods resulted in slightly different results, we believe that for deductive coding, AI-assisted methods may be a realistic option for processing large amounts of data.

Although deductive coding was similar across analysis methods, inductive analysis was less specific and more difficult to interpret because inductive codes varied across methods in both content and scope. Specifically, AI-assisted inductive codes were often multifaceted codes that included compounded or overlapping meaning and tone (i.e., Agriculture & Local Food Production), while traditional qualitative analysis identified inductive codes that were more discrete and specific (i.e., Nutrition Education). This is not surprising as reflexive content analysis emphasizes discrete, direct interpretations of the qualitative content (Nicmanis, 2024), while GPT-4 Plus and GPT for Sheets were asked to interpret community responses, which resulted in broader codes with overlapping meaning and tone. Further, AI-assisted thematic analysis was shaped by public health practitioner oversight, particularly with the refinement of themes, making it difficult to identify how much AI or the practitioner shaped the final thematic results. Ultimately, reflexive content analysis created sub-categories that were sensitive to the original meaning of the community comment, while AI-assisted analysis created generalized themes. Depending on the goal of the project, this difference (discrete and specific v. overarching and generalized) may be important to consider when choosing a method of analysis, as each could be useful in different contexts.

When examining the results of AI-assisted inductive codes, there are important limitations that should be considered if using AI-based methods for qualitative analysis. First, when considering the results of mapped comments to AI-assisted codes, some comments did not make logical sense for the code and the confidence level at which they were identified. For example, the comment “world peace” was coded within the “Sustainability & Environment” inductive code as the 23rd comment of all 229 comments mapped to these themes, representing a relatively high confidence. While the community member may have been considering sustainability and the environment in their comment, world peace may have meant something else to this community member. This highlights the limited precision of AI-assisted methods when processing large amounts of qualitative data. Another notable limitation stemming from the use of AI in this study involves the accuracy of the Post-it app, which was used to transcribe written text on Post-it notes into digital text for coding. Quality control of this transcription tool was not monitored during digitization, which might explain several illogical data points in the traditional qualitative analysis and dropped data points in the AI-assisted analysis. All comments underwent this process before qualitative analysis; thus, it is unlikely to have influenced the comparison between methods. Future studies should consider rigorously testing this method of transcription to ensure the accuracy of all data points. Finally, prompts are critical and determine what kinds of results are produced. If different prompts are used, results may vary (Bansal, 2024). Specifically, prompt engineering, or the practice of designing, refining, and optimizing prompts to guide LLMs (Ekin, 2023), has the potential to shape results and findings across studies by using carefully crafted prompts with study specific terms and definitions. Therefore, studies planning to leverage the potential of AI-assisted analysis should consider using existing best practices, such as prompt engineering frameworks to optimize prompts (Park & Choo, 2025).

To mitigate limitations of illogical coding, AI-Assisted coding in this study was completed with oversight from a public health practitioner who had extensive involvement in the larger project, development of the food plan, and participated in data collection. This oversight was essential to the methods and allowed for a critical review of AI-assisted coding results that represented the underlying beliefs, ideas, and values of the community members. Further, a known concern of AI-based models is the potential for biased coding, particularly for under-classification or misclassification of topics related to racial justice and equity (Fang et al., 2024; Hofmann et al., 2024), yet this may have been mitigated by the public health practitioners’ involvement in the data collection and equity focus of the larger project, development of a food plan. To improve AI-assisted qualitative analysis, additional training of LLMs may improve and build sufficient knowledge in the software regarding cultural, emotional, and societal implications. Through this training, the reliability of LLMs to code complicated topics such as racial justice and equity in qualitative analysis may improve. Finally, it is important to address ethical concerns related to using AI-based analysis methods with individual data. Little is known about how data entered into AI-platforms is used by the LLM or platform. This creates an ethical concern of personal or individual data being used to train the LLM or without the individual’s consent. Nevertheless, we believe that the involvement of the public health practitioner in both the data collection and oversight of the AI-analysis was critical to ensure appropriate representation of community voice, while also using AI-assisted analysis methods.

Limitations of traditional qualitative analysis were also identified. First, reliability of coding is a known concern within qualitative analysis (Noble & Smith, 2015). This study completed inter-rater reliability testing and revision, yet consistency across coders varied from 86 to 100% agreement, suggesting that human interpretation of both the codes and comments varies. Qualitative codes were also not involved in the data collection, which likely limited their ability to interpret context of community comments. Nevertheless, qualitative content analysis followed key steps to ensure a reliable result that represented key content areas of concern for the food system among the community.

Research suggests that a key benefit of using AI in many settings is the potential cost saving and increased time efficiency (Prescott et al., 2024). The study was unable to make a rigorous comparison of cost and efficiency; however, we will present key points of experience related to each method of analysis. The AI-assisted analysis required significant time for the public health practitioner to learn the software and establish a workflow for the analysis, yet after establishing a process of analysis, qualitative analysis moved quickly and was able to be used with ease in subsequent phases of the project at minimal software or program cost. Traditional qualitative analysis was time-intensive for the research team to create a codebook, code comments, and analyze data and would continue to be time intensive in each phase of analysis, likely resulting in more time overall spent on analysis. Leveraging Google Sheets did minimize software costs for both types of analysis and allowed for more seamless collaboration. This study was not able to complete a rigorous analysis of cost/time effectiveness. Given that a proposed key benefit of AI use for qualitative analysis is efficiency, future research should pay close attention to the time and cost of multiple analysis methods.

This study highlights similarities and differences in using AI-assisted and traditional qualitative methods to complete qualitative analysis, yet study limitations exist. First, this data was relatively simple in content, being that community comments were relatively short and concise, with little context; therefore, studies with more complex data may have different results when comparing AI-assisted methods to traditional qualitative analysis. Therefore, researchers may consider using AI assistance for qualitative data analysis in contexts where the data is relatively unambiguous and direct, particularly when key terms are included in prompts. Caution should be exercised when using these techniques with complex qualitative data, which might be more successful with traditional, human-assisted methods. Secondly, detailed documentation of the AI-assisted analyses was likely missed because this process was conducted in real time by a public health practitioner for the development of a food plan. Nonetheless, this study highlights how a robust analysis was completed using AI in a real application. Finally, AI platforms are changing and updating rapidly. Therefore, some of the methods in this paper may be time-bound to the platforms used (GPT for Work and GPT-4).

While differences exist across analysis methods, there are key points we would like to make about when to use AI and when to exercise caution. AI-assisted analysis performed similarly to traditional qualitative analysis in deductive coding. This was likely because the AI platforms were provided with codes and key terms for the LLM to use to organize data. Therefore, it is reasonable that AI-assisted methods could be used efficiently in public health or research settings when effective prompt engineering is used. Inductive analysis was less comparable between AI-assisted and traditional qualitative analysis. This is likely due to the interpretation of comments that AI completed during theme generation. While this is not necessarily a problem, and may be a benefit in some applications, AI did expand community comments meaning beyond what researchers saw as appropriate (e.g., World Peace was coded as Sustainability and Environment). Therefore, if AI is used to generate themes with limited prompt engineering or keywords, the results generated may be beyond the original meaning or intention of the data.

In summary, this study contributes to the evolving field of AI in prevention science by demonstrating the strengths and limitations of AI-assisted-based qualitative coding compared to traditional qualitative content analysis, while offering key considerations for use of each method. This study provides an initial framework for new evaluation practices in prevention science and public health efforts through clearly describing considerations, strengths, and limitations for using AI-assisted qualitative analysis.

## Conclusion

AI-assisted methods have the potential to rapidly process qualitative data with results that are comparable to traditional qualitative analysis, particularly in regard to overarching themes. AI-assisted analysis has limitations, but many of these can be overcome or minimized by intentional human oversight and prompt engineering. Given the need for prevention science to identify changing trends in human health and behavior to minimize future risks, the field of prevention science should consider leveraging the AI to rapidly process qualitative data that provides context to human health.

## Supplementary Information

Below is the link to the electronic supplementary material.

ESM 1 (DOCX 125 KB)

## Data Availability

Community Engagement data used for analysis are available upon request from the corresponding author and the City of Austin.

## Appendix

**Table 7:** Standards for Reporting Qualitative Research (SRQR)

Title and abstract	Pg/line #
Title—Concise description of the nature and topic of the study Identifying the study as qualitative or indicating the approach (e.g., ethnography, grounded theory) or data collection methods (e.g., interview, focus group) is recommended	1, 1–3
Abstract—Summary of key elements of the study using the abstract format of the intended publication; typically includes background, purpose, methods, results, and conclusions	2, 3–21
Introduction	
Problem formulation—Description and significance of the problem/phenomenon studied; review of relevant theory and empirical work; problem statement	3, 12–22
Purpose or research question—Purpose of the study and specific objectives or questions	4, 14–17
Methods	
Qualitative approach and research paradigm—Qualitative approach (e.g., ethnography, grounded theory, case study, phenomenology, narrative research) and guiding theory if appropriate; identifying the research paradigm (e.g., postpositivist, constructivist/interpretivist) is also recommended; rationale	8, 2–9; 12, 21–13,10
Researcher characteristics and reflexivity—Researchers’ characteristics that may influence the research, including personal attributes, qualifications/experience, relationship with participants, assumptions, and/or presuppositions; potential or actual interaction between researchers’ characteristics and the research questions, approach, methods, results, and/or transferability	13, 12–117
Context—Setting/site and salient contextual factors; rationale	5, 1–6,15
Sampling strategy—How and why research participants, documents, or events were selected; criteria for deciding when no further sampling was necessary (e.g., sampling saturation); rationale	5, 15–6,15
Ethical issues pertaining to human subjects—Documentation of approval by an appropriate ethics review board and participant consent, or explanation for lack thereof; other confidentiality and data security issues	5, 10–13
Data collection methods—Types of data collected; details of data collection procedures including (as appropriate) start and stop dates of data collection and analysis, iterative process, triangulation of sources/methods, and modification of procedures in response to evolving study findings; rationale	5, 15–6,15
Data collection instruments and technologies—Description of instruments (e.g., interview guides, questionnaires) and devices (e.g., audio recorders) used for data collection; if/how the instrument(s) changed over the course of the study	6,17–7,5 9,2–9,9
Units of study—Number and relevant characteristics of participants, documents, or events included in the study; level of participation (could be reported in results)	6,6–15
Data processing—Methods for processing data prior to and during analysis, including transcription, data entry, data management and security, verification of data integrity, data coding, and anonymization/de-identification of excerpts	6,17–7,5
Data analysis—Process by which inferences, themes, etc., were identified and developed, including the researchers involved in data analysis; usually references a specific paradigm or approach; rationale	7, 2–15,3
Techniques to enhance trustworthiness—Techniques to enhance trustworthiness and credibility of data analysis (e.g., member checking, audit trail, triangulation); rationale	12,8–17 14,19–15,4
Results/findings	
Synthesis and interpretation—Main findings (e.g., interpretations, inferences, and themes); might include development of a theory or model, or integration with prior research or theory	15, 22–20,1
Links to empirical data—Evidence (e.g., quotes, field notes, text excerpts, photographs) to substantiate analytic findings	20,3–9
Discussion	
Integration with prior work, implications, transferability, and contribution(s) to the field**—**Short summary of main findings; explanation of how findings and conclusions connect to, support, elaborate on, or challenge conclusions of earlier scholarship; discussion of scope of application/generalizability; identification of unique contribution(s) to scholarship in a discipline or field	20,3–22,17
Limitations—Trustworthiness and limitations of findings	22,19–23,23
Other	
Conflicts of interest—Potential sources of influence or perceived influence on study conduct and conclusions; how these were managed	26,15–27,22
Funding—Sources of funding and other support; role of funders in data collection, interpretation, and reporting	26, 16–19

O'Brien BC, Harris IB, Beckman TJ, Reed DA, Cook DA. Standards for reporting qualitative research: a synthesis of recommendations**.** *Academic Medicine*, Vol. 89, No. 9/Sept 2014, https://doi.org/10.1097/ACM.0000000000000388
