# Supplementary material for: Comparing AI-Assisted Coding and Traditional Qualitative Analysis: a Study Examining Differences in Methods and Results of AI-Assisted Coding and Traditional Content Coding Using Community Engagement Data Collected During the Development of a Municipal Food Plan
Source: Prev Sci. 2026 Mar 26;27(7):1163–75. doi: 10.1007/s11121-026-01904-4 (PMC13550024; doi:10.1007/s11121-026-01904-4)
Supplement: Supplementary file 1 [file 11121_2026_1904_MOESM1_ESM.docx]

Supplemental Figure 1. Flow chart of key steps in AI-assisted v. Human-center approaches of qualitative data analyses.

Supplemental Table 1. Percent agreement across coders (*n*=2) by code across both Hopes and Dreams and Challenges and Concerns during human-assisted analysis.

| **Code** | **% Agreement**  **(Hopes and Dreams)** | **% Agreement**  **(Challenges and Concerns)** |
| --- | --- | --- |
| Food Production | 86.63% | 92.49% |
| Food Consumption/Access | 84.01% | 89.45% |
| Food Markets/Retail | 96.66% | 98.57% |
| Food Waste | 92.36% | 85.87% |
| Food Processing/Distribution | 97.61% | 99.46% |
| Food Justice | 99.05% | 98.39% |
| Schools | 97.14% | 97.14% |
| Nutrition Education | 92.60% | 96.24% |
| Inclusive/Culturally Relevant Foods | 96.18% | 96.96% |
| Food Policy | 97.37% | 97.85% |
| Environmental Impact | 97.85% | 97.85% |
| Transportation | 99.28% | 97.85% |
| Food Quality | 87.59% | 90.34% |
| Health | 97.14% | 98.75% |
| Cost and Income | 94.51% | 89.09% |
| Housing | 96.66% | 96.42% |
| Community | 93.32% | 98.57% |
| Miscellaneous | 98.57% | 99.64% |
| Irrelevant | 94.75% | 96.78% |
